# Comparative Evaluation of the New HIV VIDAS® DUO AG/AB Assay With DUO Ultra on Well Characterized Participants From the HIV‐1 Acute Infection French ANRS PRIMO Cohort

**DOI:** 10.1002/jmv.70953

**Published:** 2026-05-04

**Authors:** Gilbert Mchantaf, Xavier Maia, Valérie Andriantsoanirina, Océane Dehan, Karl Stefic, Laurent Hocqueloux, Olivier Robineau, Alain Makinson, Cécile Goujard, Laurence Meyer, Véronique Avettand‐Fenoel, ANRS PRIMO cohort

**Affiliations:** ^1^ CHU d'Orléans, Virologie Orléans France; ^2^ Université d'Orléans, LI2RSO Orléans France; ^3^ AP‐HP, Hôpital Bicêtre, Santé Publique Paris France; ^4^ INSERM CESP U1018, Université Paris Saclay Paris France; ^5^ Université de Tours, INSERM U1259, CHRU de Tours Tours France; ^6^ CHU d'Orléans, service des maladies infectieuses et tropicales Orléans France; ^7^ Hôpital Guy Chatiliez Tourcoing France; ^8^ Infectious Diseases Department & INSERMU1175 Montpellier University Hospital Montpellier France; ^9^ Service de Médecine Interne et d'Immunologie Clinique, Hôpital Bicêtre, APHP Paris France

**Keywords:** agreement analysis, Fiebig classification, HIV 4TH Generation ELISA test, HIV‐1 acute infection, HIV‐1 diagnosis

## Abstract

Diagnosing HIV‐1 infection as early as the primary infection is crucial. In this context, we performed a comparative evaluation and agreement analysis of the newly proposed serological assay for the combined detection of total antibodies against HIV and P24 antigen (HIV VIDAS® DUO AG/AB, bioMérieux®), with the previously available HIV VIDAS® DUO Ultra assay in the well characterized acute‐HIV‐infection multicentric ANRS‐CO‐06‐PRIMO Cohort in classification of Fiebig stages combined with confirmatory testing. Samples collected on the day of enrollment in the cohort, between April 2024 and June 2025, were analyzed with both serological assays. We also performed a recency infection assay, an HIV‐1 westernblotting and the measurement of HIV‐RNA. Acute infection was classified according to Fiebig stages and compared between both assays. All samples (*n* = 77) were positive with both assays. One major discordance was noted, with a positive antigen and a negative antibody reaction with DUO AG/AB, with an opposite configuration with DUO Ultra. A high proportion of not determinable signals (26% to 68%) was noted with both assays, prompting a difficulty in classifying acute infection stages in the absence of confirmatory assays. The high rate of not determinable signals especially with P24 antigen with both assays demonstrated in our study, could prompt a difficulty in acute infection classification according to Fiebig.

## 1 Introduction

Diagnosing Human Immunodeficiency Virus (HIV) infection as early as possible is critical for early initiation of combined antiretroviral therapy (cART). Early cART preserves the immune system and helps control the dissemination of the infection [1, 2, 3, 4]. Early cART has also an extensive impact on HIV‐1 reservoir [5, 6] as it has been shown for both total and integrated HIV‐1 DNA in comparison with treatment initiated during the chronic stage [1, 5, 7, 8, 9, 10]. Thus, having tools that enable early diagnosis and accurate identification of the acute stages of infection is crucial for selecting potential candidates for future cure strategies.

Stages of acute HIV‐1 infection are usually defined according to Fiebig classification. This widely used classification in cure trials is based on the kinetics and the dynamics of P24 antigen, anti‐HIV‐1 antibodies and HIV‐1 plasma viral load [11]. Fiebig I is defined as the sole positivity of HIV‐1 RNA in circulating blood, while Fiebig II is defined by the positivity of P24 antigen and the lack of detectable antibodies in sera. Fiebig III is defined by the positivity of both P24 antigen and antibodies with immunoassays with a negative detection of specific antibodies with HIV‐1 westernblotting. Fiebig IV is defined by indeterminate anti‐HIV‐1 profile with westernblotting. Fiebig V is defined with a positive but incomplete westernblotting (lacking anti‐p31/34), while Fiebig VI is an open‐ended stage, characterized with a full antibody profiling with westernblotting (i.e., the positivity of anti‐p31).

Laboratory diagnosis of HIV‐1 infection is based on a reactive fourth generation, combined/mixed HIV ELISA test. These tests allow both the detection of the HIV‐1 P24 antigen and the antibodies against HIV‐1/HIV‐2. While most commercially available HIV ELISA tests do not separate signals from antigen reactivity and antibody reactivity, HIV VIDAS DUO tests allow a differentiation of antigen and antibodies signals. Recently in 2024, Biomerieux® launched a new serological test (VIDAS® HIV DUO AG/AB) in place of the previous one, with a lower limit of detection of P24 antigen (0.33 UI/mL) than the previously available kit VIDAS® HIV DUO Ultra (0.5 UI/mL) (manufacturer documentation). This better analytical sensitivity could lead, in theory, to an earlier diagnosis of HIV‐1 infection than the previous test. This test has a CE‐IVD marking in Europe but is not yet FDA approved. Here, we aimed to test the analytical agreement between this new assay and VIDAS® HIV DUO Ultra in a well characterized French primary infection cohort (ANRS‐PRIMO CO06) to define the stage of acute infection.

## 2 Materials and Methods

### 2.1 Study Population

The ANRS‐MIE (Agence Nationale de Recherches sur le SIDA et les Hépatites Virales, Maladies Infectieuses Emergentes) PRIMO CO6 cohort is an ongoing French national cohort that enrolls People‐Living‐With‐HIV‐1 (PLWH), presenting with acute or early HIV‐1 infection and are not yet treated with any cART, defined by: (1) Positive plasma HIV‐1 RNA or a positive P24 antigen and a negative 4th generation ELISA test in the past 6 weeks prior to inclusion, (2) Positive plasma HIV‐1 RNA or a positive P24 antigen and a positive 4th generation ELISA test with Ag+/Ab− in the past 6 weeks prior to inclusion, (3) Positive plasma HIV‐1 RNA or a positive P24 antigen and a positive 4th generation ELISA test associated with a negative or incomplete westernblotting or immunoblotting in the past 6 weeks prior to inclusion, or (4) a positive ELISA test with a negative screening test in the past 3 months. The ANRS‐PRIMO cohort was approved by the regional ethics committee (CPP Ile de France III) (ClinicalTrials.gov ID NCT03148964) and all participants have given their written consent. Participants enrolled between April 2024 and June 2025 were included in this study. Frozen biological samples taken at inclusion in the cohort (i.e., at primary infection) were analyzed.

### 2.2 Analytical Methods

The following analyses were centralized in the laboratory of virology of Centre Hospitalier Universitaire d'Orléans.

VIDAS® HIV DUO AG/AB assay is a qualitative automated test for the combined detection of total antibodies against HIV‐1 and HIV‐2 and P24 antigen with differentiation of antigen and antibodies signals using ELFA technique (Enzyme‐linked fluorescent assay). An RFV (Relative Fluorescence Values) equal or greater than 1 is considered as positive. According to the manufacturer's insert sheet, a very high RFV for antibodies could mask the reactivity of antigen and vice‐versa. In this case, a not determinable (ND) signal appears for the antibodies or antigen associated with a positive interpretation of the other parameter. According to the manufacturer's information sheet, the limit of detection (LOD) announced for this test is 0.33 UI/mL for antigen P24 and below 0.5 UI/mL for antibodies. The estimated duration of the test's workflow is 88 min.

VIDAS® HIV DUO Ultra (HIV5) is also an automated 4th generation HIV test with a differentiation of antibodies and antigen signals that is based on the same technology as HIV DUO AG/AB assay. An RFV equal or greater than 0.25 is considered as positive with an LOD of 0.5 UI/mL for P24 antigen. The duration of the test's workflow is estimated at 123 min.

HIV specific antibodies profiling with westernblotting was performed using NEW LAV BLOT I (Bio‐rad®) on serum. Westernblotting results were interpreted according to the World Health Organization (WHO) criteria. Plasma viral load was quantified using cobas® HIV‐1 Test on cobas 4800 (Roche®).

We used Fiebig's classification to classify the stages of acute HIV infection as described in the introduction [11].

A centralized HIV‐1 infection EIA recency test was realized in HIV national reference center at the Centre Hospitalier Universitaire de Tours as previously described [12]. An index below 0.5 was interpreted as a recent infection of fewer than 180 days.

### 2.3 Statistical Analysis

Continuous variables were expressed as median with its interquartile range (IQR). For categorical variables (ND vs. determinable), McNemar's test (Chi square test for paired data) was used (test accessible at https://www.graphpad.com/quickcalcs/mcnemar1/). Agreement between both assays for the results of antibodies and P24 antigen were compared using Cohen kappa (test accessible at https://www.graphpad.com/quickcalcs/kappa1/).

## 3 Results

### 3.1 Study Population

In total, 77 participants from ANRS PRIMO cohort were included (Table 1). They were all untreated, i.e., cART naïve at inclusion. The median HIV‐1 plasma VL was 5.5 (IQR 4.5; 6.3) log copies/mL. HIV‐1 antibodies profiling with westernblotting was incomplete for most participants with a median of 6 antibodies (3; 9). The median HIV‐1 infection recency test was of 0.02 (0.01; 0.04) in favor of a recent infection. One participant had a recency test value greater than 0.5, however, he presented with signs of primary infection 2 weeks before enrollment, with an incomplete immunoblot (only presence of antibodies against gp160 and gp41) on inclusion day prompting his inclusion in the cohort. This participant had a negative HIV ELISA screening test 6 months prior.

**Table 1:** Study participants clinical and virological characteristics.

	*N* = 77
Plasma HIV‐1 RNA (log copies/mL)	5.5 (4.5; 6.3)^a^
CD4 T cells count (per mm^3^)	512 (307; 687)^a^, ^b^
Fiebig stages *n* (%)	
I	0 (0%)
II	6 (8%)
III	0 (0%)
IV	28 (36%)
V	13 (17%)
VI	30 (39%)^c^

^a^ Continuous variables are expressed as median (with interquartile range IQR).

^b^ Missing data for three participants.

^c^ These participants met the inclusion criteria of the cohort at the time of infection's diagnosis and completed their western blotting profile at the time of inclusion.

### 3.2 VIDAS® HIV DUO Ultra and VIDAS® HIV DUO AG/AB Agreement Analysis

All the tested samples (*n *= 77) were positive with both serological assays. The repartition of interpretation of RFVs is detailed in Table 2. The proportions of ND signals for P24 antigen with HIV DUO AG/AB and HIV DUO Ultra were respectively 68% and 60% (McNemar's test *p* = 0.11). These proportions were respectively 29% and 26% for ND anti‐HIV signals with HIV DUO AG/AB and HIV DUO Ultra (McNemar's test *p* = 0.68).

**Table 2:** Agreement in antibodies and P24 antigen results between VIDAS® HIV DUO AG/AB and VIDAS® HIV DUO Ultra.

	VIDAS HIV DUO AG/AB					
	P24 Ag+/Anti‐HIV−	P24 Ag+/Anti‐HIV ND	P24 Ag+/Anti‐HIV+	P24 Ag−/Anti‐HIV+	P24 Ag ND/Anti‐HIV+	Total
**VIDAS HIV DUO Ultra**						
**P24 Ag+/Anti‐HIV−**	1	0	0	0	0	1
**P24 Ag+/Anti‐HIV ND**	0	18	0	0	2	20
**P24 Ag+/Anti‐HIV+**	0	3	0	0	0	3
**P24 Ag−/Anti‐HIV+**	1	0	0	0	6	7
**P24 Ag ND/Anti‐HIV+**	0	1	1	0	44	46
**Total**	2	22	1	0	52	77

+, positive; −, negative; Ag, antigen; ND, not determinable.

P24 antigen reporting agreement between both assays was judged as substantial agreement with a Cohen's kappa *k* = 0.712 (standard error ± 0.075) (Table 3). Similarly, the agreement on anti‐HIV antibodies reporting between both assays was also judged as substantial (*k* = 0.792 ± 0.076) (Table 4). One major discordance between both assays was found for a participant with a positive antigen signal (1.08 for a cut‐off of 1) and negative antibodies reactivity (0.31 for a cut‐off of 1) with VIDAS® HIV DUO AG/AB and a negative antigen signal (0.13 for a cut‐off of 0.25) and a positive antibodies signal (0.26 for a cut‐off of 0.25) with VIDAS® HIV DUO Ultra (Table 2). Indeed, HIV DUO Ultra results would classify the participant's acute infection at Fiebig III at least, while HIV‐1 DUO AB/AG results would classify the acute infection at Fiebig II. Of note, HIV‐1 westernblotting was indeterminate with only anti‐P24 and anti‐p18 low positive bands, prompting a Fiebig IV classification for this participant.

**Table 3:** Agreement analysis for Ag P24 reporting between VIDAS® DUO AG/AB and VIDAS DUO Ultra.

	VIDAS HIV DUO AG/AB			Total
	Ag P24+	Ag P24−	Ag P24 ND	
VIDAS HIV DUO Ultra				
Ag P24+	22	0	2	24
Ag P24−	1	0	6	7
Ag P24 ND	2	0	44	46
Total	25	0	52	77

+, positive; −, negative; Ag, antigen; ND, not determinable.

**Table 4:** Agreement analysis for Anti‐HIV antibodies reporting between VIDAS® DUO AG/AB and VIDAS® DUO Ultra.

	VIDAS HIV DUO AG/AB			Total
	Anti‐HIV+	Anti‐HIV−	Anti‐HIV ND	
VIDAS HIV DUO Ultra				
Anti‐HIV+	51	1	4	56
Anti‐HIV−	0	1	0	1
Anti‐HIV ND	2	0	18	20
Total	53	2	22	77

+, positive; −, negative; ND, not determinable.

Moreover, among ND P24 antigen signals with HIV VIDAS® DUO Ultra, two positive signals were detected with DUO Ag/Ab (index of 26.28 and 4.68). Similarly, two ND P24 antigen signals with HIV DUO Ag/Ab were positive for Ag P24 with HIV DUO Ultra (3.7 and 5.9) and five were negative.

Finally, considering serological assay results combined with westernblotting and HIV‐1 RNA results, no difference in Fiebig classification was observed, regardless the VIDAS® DUO version used as Fiebig classification of early infection stages were adjusted with westernblotting.

## 4 Discussion

Early diagnosis of HIV‐1 infection offers many opportunities for PLWH. The earlier treatment is initiated, the greater the impact on limiting the risk of HIV transmission and the establishment of HIV‐1 reservoir [1, 8, 13]. Accurately classifying acute infection stages is also important for the selection of PLWH that would be suitable for cure trials, as these PLWH are the best candidate for such strategies [14]. Indeed, early treated PLWH have a greater chance of achieving sustainable virological control after treatment interruption than those treated during the chronic infection [14, 15, 16, 17, 18, 19, 20]. It is in this context, that we evaluated the newly proposed HIV VIDAS® DUO AG/AB serological kit test by bioMérieux® in the classification of stages of early HIV‐1 infection in a cohort of well characterized PLWH diagnosed at the primary infection. We also compared the agreement between this assay and the now discontinued serological test HIV VIDAS® DUO Ultra (bioMérieux®). Both tests allow for a separate detection of HIV‐1 P24 antigen and antibodies against HIV‐1 thus permitting recognition of early stages of HIV‐1 infection.

Our results indicate that this new VIDAS® DUO AG/AB is suitable for the diagnosis of early infection as it was positive for all tested samples including participants enrolled at Fiebig II stage. One major discordance was noted between both assays, where a positive antigen and a negative antibody reaction was noted with VIDAS® DUO AG/AB, while an opposite configuration was noted with VIDAS Ultra. Our results indicate that these tests may not be sufficient nor suitable to stage acute infection according to Fiebig. The high proportion of Not determinable signals of antigen or antibodies, reflecting a too high signal with one parameter masking the potential reactivity of the other, does not impact HIV‐1 infection diagnosis. However, this high proportion for P24 antigen with both tests could complicate the classification of early infection stages. In addition, a not determinable signal of antibodies could lead to a false classification of participants as Fiebig II stage. Given the high frequency of ND signals, accurate staging in this study relied on the combination of serological assays results, westernblotting and plasma viral load results. Furthermore, the absence of Fiebig III cases in this study may be related to the possibility, that when the P24 antigen is positive, antibodies signals could be masked and reported as ND, resulting in misclassifying participants as Fiebig II instead of Fiebig III. It is therefore useful for bioMérieux® to change the configuration of the HIV‐1 VIDAS® DUO kit, aiming to minimize not determinable signals for positive samples and therefore ensuring a higher rate of accurate positive and negative results.

One of the limitations of our study is the lack of participants included at Fiebig I which would have allowed us to specifically evaluate the performance of these tests at this stage. The high proportion of participants included at late stages of acute infection and the low proportions of participants included at Fiebig II, limit the assessment of the assay's performance in early stages of HIV‐1 infection. However, access to these kinds of samples is difficult to obtain for HIV testing validation. In addition, CD4 data were missing for a small number of participants, however, this did not impact the interpretation of our results.

In conclusion, our results in mainly late acute/early serconversions demonstrate that this new HIV VIDAS® DUO AG/AB assay can be suitable for the diagnosis of HIV‐1 infection at acute stage in a well characterized HIV‐1 acute infection cohort. We highlighted the inconsistency in reporting positive signals especially of P24 antigen, a crucial element for classification of early infection: interpretation of not determinable signals for antigen P24 or antibodies in PLWH in case of suspected primary infection should therefore be precautious. A consolidation with antibodies profiling with HIV‐1 westernblotting and the levels of replication appreciated with HIV‐1 plasma viral load is thus crucial for identification and classification of acute infection.

## Conflicts of Interest

VAF has received institutional grants from ViiV Healthcare and honoraria and travel grants from ViiV Healthcare and Gilead Sciences for participation in educational meetings and conferences. LH has received institutional grants from GILEAD Sciences and ViiV Healthcare, honoraria and travel grants from Gilead Sciences, ViiV Healthcare, and MSD for participation in Adboards and conferences. This study was funded by the ANRS‐MIE.

## Data Availability

The data that support the findings of this study are available from the corresponding authors® upon reasonable request.
